# Perioperative chemotherapy in locally advanced gastric cancer in Chile: from evidence to daily practice

**DOI:** 10.3332/ecancer.2021.1244

**Published:** 2021-06-07

**Authors:** Bettina Müller, Carlos García, José A Sola, Wanda Fernandez, Patrick Werner, Mauricio Cerda, Jeannie Slater, Carlos Benavides, Jorge Arancibia, Rodrigo Ascui, Felipe Reyes, Mary Ann Stevens, Juan Pablo Miranda, Martin Buchholtz, Alejandro H Corvalan

**Affiliations:** 1 Medical Oncology Department, Instituto Nacional del Cáncer, Profesor Zañartu 1010, 8380455 Santiago, Chile; 2 Chilean Cooperative Oncology Group (GOCCHI), José Manuel Infante 125, Oficina 11, 7500641 Santiago, Chile; 3 Digestive Surgery Department, Hospital San Borja Arriarán, Santa Rosa 1234, 8360160 Santiago, Chile; 4 Department for Medical Oncology, Clinica Alemana de Santiago, Vitacura 5951, 7650568 Santiago, Chile; 5 Pathology Department, Hospital San Borja Arriarán, Santa Rosa 1234, 8360160 Santiago, Chile; 6 MOLIT Institute Heilbronn, Im Zukunftspark 10, 74076 Heilbronn, Germany; 7 Integrative Biology Program, Institute of Biomedical Sciences, Center for Medical Informatics and Telemedicine, Faculty of Medicine, Universidad de Chile, Independencia 1027, 8380453 Santiago, Chile; 8 Biomedical Neuroscience Institute, Independencia 1027, 8380453 Santiago, Chile; 9 Pathology Department, Clinica Alemana de Santiago, Vitacura 5951, 7650568 Santiago, Chile; 10 Medical Oncology Department, Hospital Naval Almirante Nef, Valparaiso, Alessandri s/n, 2520000 Viña del Mar, Chile; 11 Medical Oncology Department, Hospital Regional de Concepción, San Martín 1436, 4070038 Concepcion, Chile; 12 Medical Oncology Department, Hospital del Salvador, Salvador 364, 7500922 Santiago, Chile; 13 Surgery Department, Instituto Nacional del Cáncer, Profesor Zañartu 1010, 8380455 Santiago, Chile; 14 Hematology and Oncology Department, Advanced Center for Chronic Diseases (ACCDiS), Pontificia Universidad Católica de Chile, Portugal 61, 8330034 Santiago, Chile

**Keywords:** stomach neoplasms, survival rate, laparoscopy, public health, feasibility studies

## Abstract

Gastric cancer (GC) is a leading cause of cancer death in Chile. Although recommended in international guidelines since 2006, perioperative chemotherapy was not available to patients in the public health system in Chile until 2016. We conducted an observational study to assess the feasibility of this strategy in public hospitals in Chile (Observational Study of Perioperative Chemotherapy in Locally Advanced Gastric Cancer – PRECISO). Patients with locally advanced, operable GC were offered to receive preoperative chemotherapy with Epirubicin + Cisplatin + Capecitabine (ECX) for three cycles followed by curative surgery. Staging included abdominal CT scan and laparoscopy if peritoneal carcinomatosis was suspected. Postoperative ECX for three cycles was recommended. Between August 2010 and March 2013, 110 patients were screened and 61 enrolled. Median age was 62 years (23–76 years) and most patients had good performance status at baseline (Eastern Cooperative Oncology Group performance status score (ECOG) 0: 42, ECOG 1: 19). Tumour site was proximal in 32 (52%) and medial and distal in 29 (48%) patients. All but four patients (*n* = 57, 93%) completed three cycles of preoperative chemotherapy. Fifty-six patients were operated and 54 (89%) had a curative resection. Thirty-three patients (54%) had pT0-2, and 18 (30%) had pN0 tumours, with two patients achieving a complete response. As of 20 December 2020, 39 patients died, 32 due to GC, one within 30 days of surgery, two due to intestinal obstruction at 5 and 3 months after surgery and four due to other causes. Five-year survival rate was 38%. We conclude that perioperative chemotherapy is feasible in public hospitals in Chile and should be offered to patients with locally advanced GC.

## Background

Worldwide, gastric cancer (GC) is one of the leading cancers in incidence, being fourth and seventh in males and females, respectively. GC represents the third (males) and fifth (females) most common cause of cancer deaths globally [1]. Despite the decline in age-standardised incidence and mortality rates due to GC in the last decades, given changes in age distribution and population growth, especially in low- and middle-income countries, the absolute numbers of new cases and deaths are rising [2]. Chile is the Latin American country with the highest incidence of GC, with an estimated age-standardised incidence in 2018 of 17.8. Every year, more than 5,000 persons are diagnosed with GC in Chile, representing a major public health problem [1].

At the time of diagnosis, the majority of patients present locally advanced or metastatic disease, while early lesions are diagnosed in less than 10% of cases [3]. Surgery achieving R0 resection is the cornerstone for a curative treatment, but even in high volume centres, the curative resection rates are low (62.4%); and 5-year survival rates after surgery are dismal, as shown in a series of 423 patients from a single institution with 33% of all patients operated being alive at 5 years [4].

To improve cure rates, different multimodality treatments have been studied, establishing adjuvant chemoradiation [5], adjuvant chemotherapy [6] and perioperative chemotherapy as standard treatments for GC [7, 8]. Postoperative treatments are difficult to start and to deliver, as was shown in our experience with adjuvant chemoradiation, with a median time from surgery to treatment initiation of 14 weeks (range: 4.6–30.1 weeks) and only 72% of patients completing the planned treatment [9].

In 2006, the Chilean national cancer action plan included GC in the list of prioritised diseases within the Explicit Guarantees System [10]. However, only surgery was included in the plan, establishing a timeframe of 30 days from diagnosis during which the patient should receive surgery [11].

Additional barriers for preoperative treatments in GC were: (i) lack of reimbursement for chemotherapy costs, (ii) patients were often presented at the multidisciplinary tumour board after surgery had already been performed and (iii) surgeons were concerned about progressive disease while on chemotherapy or an increase in surgical morbidity after chemotherapy, made it nearly impossible to offer a preoperative treatment strategy to patients in the public health system in Chile.

To provide data on the feasibility of perioperative chemotherapy in Chile, we conducted a prospective, observational study to evaluate the efficacy and safety of perioperative chemotherapy with the ECX regimen (Epirubicin + Cisplatin + Capecitabine) at public hospitals (Observational Study of Perioperative Chemotherapy in Locally Advanced Gastric Cancer (PRECISO) – GOCCHI-2009-01 – NCT0163320).

## Methods

### Participants

Patients referred for neoadjuvant chemotherapy were screened. Patients older than age 18 diagnosed with histologically proven, locally advanced gastric carcinoma (cT3 or 4 and/or cN+), with no evidence of distant metastasis (cM0), and with an operable tumour, were eligible for this study. The 6th Edition of the American Joint Committee on Cancer (AJCC) Cancer Staging Manual was used [12]. The resectability of the tumour was confirmed by a multidisciplinary tumour board or a specialised surgeon. Performance status of ECOG 0–1 [13], and an adequate haematological, hepatic and renal function (including creatinine clearance > 60 mL/minute calculated using the Cockroft–Gault equation) were also required. Patients with uncontrolled diarrhoea, inability to swallow capecitabine tablets, cardiac dysfunction, severe dysphagia or gastric retention with a caloric intake of <1,500 kcal/day were excluded. Standard staging procedures included abdominal CT, chest radiography or thoracic CT and laparoscopy (required if suspicion for peritoneal spread). HER 2 testing was done at a central laboratory on a subgroup of patients who gave explicit consent for experimental HER 2 testing and for whom a sample could be retrieved and analysed. The Quality-of-Life Sub-study will be presented in a subsequent publication.

The protocol was approved by the Ethics Committee of the Northern Metropolitan Health Service and all patients signed the approved informed consent form prior to treatment. All patients who signed the consent form and met all eligibility criteria were enrolled in the study. The chemotherapy drugs Epirubicin and Cisplatin for the preoperative treatment were donated by LKM, and the Instituto Nacional del Cáncer (Incancer) provided Capecitabine.

### Treatment and follow-up

Patients received preoperative chemotherapy with Epirubicin (LKM) 50 mg/m^2^ IV Day 1 + Cisplatin (LKM) 60 mg/m^2^ IV Day 1 + Capecitabine (Xeloda®) 1,250 mg/m^2^ orally (PO) Day 1–21 (ECX) every 21 days for three cycles. Treatment delays and dose reductions were standardised. Curative surgery with total or subtotal gastrectomy and D2 lymphadenectomy was planned 3–6 weeks after the last dose of chemotherapy. Postoperative chemotherapy with the same regimen was recommended, but the medication could not be provided free of charge to the patients. After completion of perioperative treatments, patients were followed at months 6 and 12 after surgery and then yearly, with clinical evaluation. Imaging studies or endoscopy was performed on suspicion of progression.

### Assessment of efficacy

The primary endpoint was downstaging defined as the proportion of patients having T<3 tumours or N0 disease after three cycles of preoperative chemotherapy. Initially, downstaging was planned to be done by imaging, comparing baseline CT scan with a second CT scan done before surgery, but given the limitation of staging by CT imaging, final analysis for T and N stage was done based on pathological staging. Toxicity was a secondary endpoint and was assessed at day 1 of every chemotherapy cycle and prior to surgery using the CTCAE v3.0 manual [14]. Response rate measured by RECIST could not be assessed for the T and N stage, given the difficulty to obtain measurable lesions using CT scans. Surgical mortality rate was defined as patients deceased for any cause within 30 days of surgery. Compliance with postoperative chemotherapy was defined as the number of patients who started postoperative chemotherapy after curative resection and the number of patients who completed three cycles. Overall survival was calculated from the date of enrolment to death. Patients alive were censored at the time of last follow-up. Vital status for patients with no follow-up visit at the clinic was obtained by consultation of the civil registry. Patients not recorded as deceased were assumed alive and censored at the time of consultation of the civil registry. Death data were obtained through death certificates delivered by the civil registry.

### Statistical analysis and data management

Source data were documented in the clinical charts at the Incancer using specially designed templates to ensure the completeness of data documentation for patient eligibility and clinical visits. Other source documents like signed informed consent forms, radiology and pathology reports were included in the patient files. Anonymised data were entered on electronic Case Report Forms (eCRFs) using a Good Clinical Practice (GCP), 21 CFR Part 11 compliant platform OpenClinica©. The eCRFs were designed by the study researchers and organised into clinical events, each with a single repetition (e.g. baseline, diagnosis, surgery). The use of eCRFs prevents data entry errors, allows one to easily keep track of form changes, helps solve data entry discrepancies and improves overall clinical team coordination. After data entry and review, reports were extracted from OpenClinica© as spreadsheets, and Matlab [15] was used to automatically compute descriptive statistics. Descriptive statistics were used for demographics baseline characteristics, TNM and toxicity evaluation.

Kaplan–Meier curves for overall survival were generated. To assess statistical differences between variables, the log-rank test was used (*p*-value < 0.05). For univariate survival analysis, the Cox proportional hazards model was applied. Statistical analysis was performed using Stata v15.0.

## Results

### Patient and tumour characteristics

**Figure 1.**
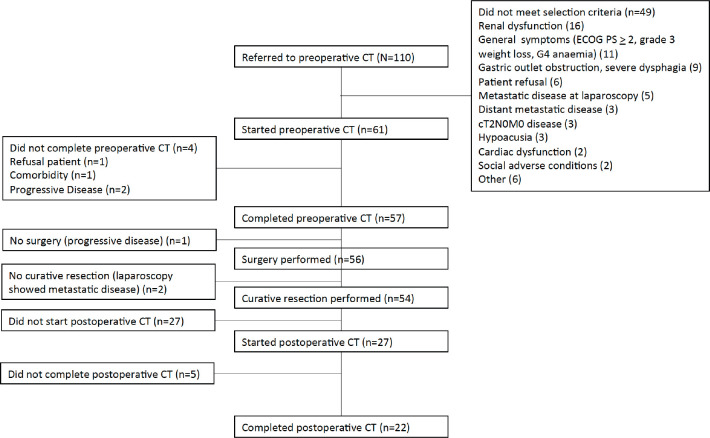
CONSORT diagram showing patients referred, reasons for exclusion (more than one criteria could be present for one patient), causes for not completing preoperative chemotherapy (CT) or surgery and reasons for not starting or completing postoperative CT.

Between August 2010 and March 2013, 110 patients were screened, and 61 were enrolled. The reasons for exclusion are shown in the CONSORT diagram (Figure 1). Creatinine clearance calculated using the Cockcroft–Gault Equation of less than 60 mL/minute was the most frequent exclusion criteria. Patient and tumour characteristics are shown in Table 1. Median age was 62 years and more than two thirds were male. Most patients (69%) presented with ECOG performance status 0 at baseline. The most frequent comorbidities were hypertension, diabetes and obesity.

All patients had a histological diagnosis by biopsy obtained during oesophagogastroduodenoscopy, and an abdominal CT scan for staging. About half of the patients had a diagnostic laparoscopy performed, ruling out a peritoneal spread. The median time from endoscopic diagnosis to enrolment was 58 days (range: 14–260 days), and the median time from staging CT to enrolment was 31 days (range: 5–83 days).

In approximately half of the patients, the tumour involved the proximal portion of the stomach and/or the gastro-oesophageal union, while the other half of patients had non-cardia GCs. One third of patients had the presence of signet cells reported on the diagnostic biopsy. Almost two thirds of the patients had an intestinal, and only 15% a diffuse histological type. Given that many pathologists used the classification of the WHO, papillary, tubular and mucinous adenocarcinomas were classified as intestinal type, whereas signet-ring cell carcinomas were classified as diffuse type. Mixed carcinomas, carcinomas and adenocarcinomas not otherwise specified (NOS) were classified as indeterminate type.

HER-2 status by immunohistochemistry could be obtained for 23 patients at baseline. In four cases (17%), overexpression of HER2 receptors was observed, one case (4%) was equivocal and 18 cases (78%) were negative.

### Treatment

Most patients (43 patients, 70%) were referred from the Hospital San Borja Arriarán, other referring centres were the Hospital San José (nine patients), the Incancer (eight patients) and the Hospital Rancagua (one patient). Chemotherapy was administered for all patients at the Incancer, while surgery was performed at the referring centre. All but four patients (*n* = 57, 93%) completed three cycles of preoperative chemotherapy: one patient refused chemotherapy after the first cycle, one patient presented a cerebrovascular accident after the second cycle and two patients progressed. One patient had progressive disease after three cycles of chemotherapy and was not submitted to surgery.

Fifty-six patients (92%) were operated on. Details of the type of surgery performed are shown in Table 2. All but two (*n* = 54, 96% of patients operated) had a curative resection, with R0 resection being confirmed in 51 (91% of patients operated). The median time from enrolment to surgery was 16 weeks (range: 11–30 weeks). Total gastrectomy was the most frequently performed surgery and the minority of patients had a splenectomy and/or pancreatectomy. Of 54 patients with curative resection, two had less than 15 lymph nodes (LNs) resected (4%), and the majority of patients (78%) had more than 25 LNs resected. One patient died within 30 days of surgery because of septic shock (1.8% surgical mortality).

After surgical resection, 27 patients started postoperative chemotherapy (50% of resected patients). Twenty-two patients (81% of the patients who started adjuvant chemotherapy) completed the planned three cycles, and five patients stopped early, three after one cycle and two after two cycles.

Toxicity for pre and postoperative chemotherapy is shown in Table 3. Although all patients experienced at least one adverse event, most of the toxicities seen were grade 1 or 2. The most frequent grade 3 or 4 toxicity observed was neutropenia, none of which was febrile neutropenia. A greater proportion of patients receiving postoperative chemotherapy presented grade 3 and 4 neutropenia compared to preoperative chemotherapy (20% versus 48%).

### Downstaging and survival

Clinical staging and pathological staging details are shown in Table 4. In 21 patients, the tumour, although locally advanced at endoscopy, could not be visualised clearly on CT scan at baseline and was staged as Tx. Enlarged LNs were reported in 38 patients (62%), while in eight cases no LNs were described (13%). In 15 cases (25%), although LNs were mentioned, these did not meet the criteria for clear N+ disease and were classified as Nx.

Pathological TNM staging revealed tumours not penetrating the serosa or beyond (ypT0–2) in 33 patients (61% of resected patients) and 18 patients (33% of resected patients) who had no LNs involved after neoadjuvant chemotherapy (ypN0). A complete pathological response (ypT0N0) was found in two patients.

As of 20 December 2020, 22 patients were still alive, 32 patients died due to the disease and 7 patients died due to other causes.

**Figure 2.**
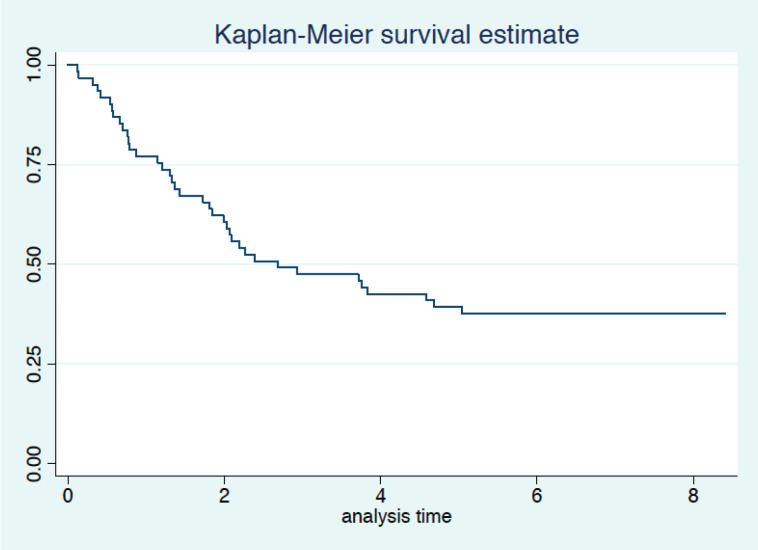
Overall survival (years from date of inclusion to date of death or last follow-up).

**Figure 3.**
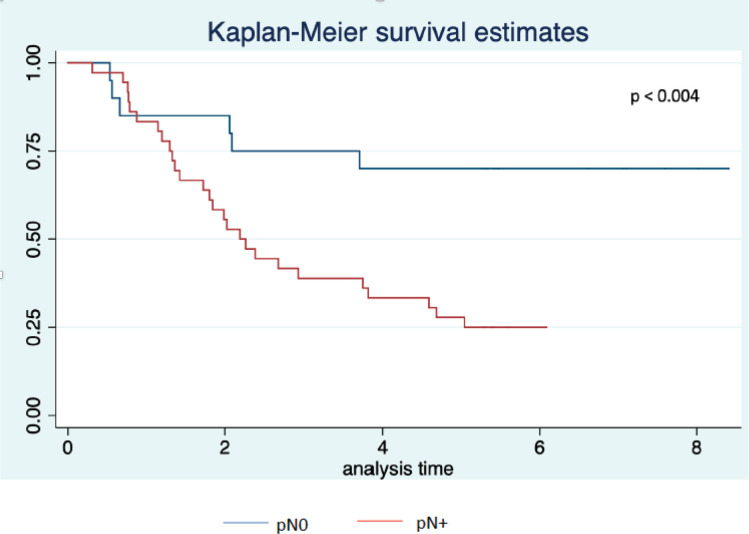
Overall survival by pN status (pN0 versus pN+) (years from date of inclusion to date of death or last follow-up).

Twenty-three patients completed the planned 5 years of follow-up. One of them was alive with recurrence, and 22 had no evidence of disease recurrence at 5 years. Median survival was 2.7 years with 3- and 5-year survival rates of 46% and 38%, respectively (Figure 2). Univariate analysis is presented in Table 5. We did not observe a prognostic impact of a performed laparoscopy, presence of signet cells or clinical T or N staging. Patients with an absence of LN involvement at surgery (pN0) had significantly better survival than patients with LN metastasis (ypN+) (Figure 3).

## Discussion

Our study shows a high proportion of patients achieving ypT0–2 tumours in 61% and yN0 disease in 33% of those who underwent curative resection. These findings are in line with the results reported by Cunningham *et al* [7] from the MAGIC trial, where ypT0–2 of 52% and ypN0 of 31% had been reported in the experimental arm. Two patients have a pathological complete response (pCR), which is comparable to the findings reported by Ychou *et al* [8], with ypT0 observed in 3% of the patients.

A complete pathological response could be further improved with the FLOT regimen, with complete regression seen in 17% of the patients [16]. The role of pCR as a prognostic factor is still to be elucidated, given the findings from the MAGIC trial where ypN0 was the only independent factor correlated with better overall survival [17]. Our findings support this observation, since of the 18 patients without LN metastasis at resection, 14 (78%) were alive in December 2020, compared to 8 (22%) of 36 resected patients with LN metastasis on the pathology report.

After completing the 5 years follow-up period for all living patients, the overall 5 years survival for our cohort is 38%. This finding again is in line with the results reported by Cunningham *et al* [7], who showed a 5-year survival rate of 36.3% in the chemotherapy arm. The FLOT regimen showed an even better overall survival compared to the Epirubicin-Cisplatin-5-Fluorouracil (ECF)/ECX regimen with an estimated 5-years overall survival rate of 45% [18]. Compared to our data for surgery followed by chemoradiation, with 5-year survival rates of 37.5% [9], we obtained similar results in a less selected cohort, since inoperable patients or patients with R1 resections were not excluded in our study, while these patients would not have received adjuvant chemoradiation.

We observe that the vast majority of the patients (92%) complete the preoperative treatment and undergo surgery. Patients progressing during chemotherapy represent a group of patients with very poor prognosis, and the preoperative treatment can select patients who will not benefit from an aggressive intervention.

Our R0 resection rate (91%) is similar to the 91% R0 resection rate reported by Biffi with preoperative chemotherapy [19]. A meta-analysis reported an improved R0 resection rate with neoadjuvant chemotherapy in GC (HR: 1.38, *p* = 0.01) [20].

Surgical mortality is often documented as death within 30 days of surgery. A Chilean series of 1,066 patients showed a decrease in this parameter in recent years, with mortality rates of 1.8% in 219 patients operated between 2011 and 2014 [21]. These numbers are similar to our findings with one death within 30 days of surgery (1.8%).

Postoperative chemotherapy is difficult to deliver, given the frailty of these patients after gastrectomy; and the number of patients completing the planned treatment is consistently low throughout the different trials, ranging between 60% [22], 50% [7] and 34% [19], similar to our findings, with 41% of the patients completing the planned three cycles.

This finding suggests that, if multimodality treatment is planned, giving systemic therapy first will give the benefit of more than one treatment strategy to more patients compared to primary surgery. Our cohort included a slightly higher proportion of patients with involvement of the cardias or upper third of the stomach, compared to the series of the main referring centre [4] with surgery as primary treatment. A selection bias towards patients with technically more challenging disease where downstaging could improve resectability cannot be ruled out.

In our study, of 110 patients screened, 49 (45%) did not meet the inclusion criteria, one third because of a calculated creatinine clearance of less than 60 mL/minute. Kuhnle *et al* [23] recently reported their experience with perioperative chemotherapy in the real world setting and did not exclude patients with renal glomerular filtration rate between 30 and 60 mL/minute. With the less nephrotoxic regimen FLOT, this could be a strategy to offer more patients a preoperative systemic treatment.

Our study has several limitations. We could not determine the clinical response after three cycles of preoperative chemotherapy, given the difficulty for accurate TNM staging by CT scan, particularly because CT scans were performed in different centres without standardised protocols. But even with such protocols, the accuracy of radiologic T and N staging after neoadjuvant chemotherapy is low [24]. Only half of the patients received staging laparoscopy, as this procedure was only mandatory in the case of suspicion; thus, subclinical spread could have been missed at the time of enrolment. Actually, one patient progressed with peritoneal carcinomatosis during preoperative chemotherapy. Surgical morbidity and progression-free survival could not be determined, as many patients were not operated on and followed at the Incancer. It is noteworthy that all pathology reports were reviewed by the principal investigator, and the date and cause of death of all deceased patients were confirmed by death certificate. Given the long follow-up, and the dismal prognosis in case of progression, we consider the overall survival data the most informative.

HER-2 testing was done only in a subset of 23 patients, and no complementary FISH assay could be performed for two cases with equivocal findings. Interestingly, three of four patients with HER2 immunohistochemistry (IHC) 3+ staining are still alive, thus raising the question about the prognostic impact of HER2 positivity in GC treated with perioperative chemotherapy, as these patients were excluded in the recent report by Martinez Lago [25].

This study provides important information to the public health authorities, supporting the decision to mandate financial coverage for perioperative chemotherapy by the public and private health insurance since 2016. Additionally, it provides reassuring data for the surgical field in Chile, leading to increasing support by surgeons for this treatment strategy.

Since 2020, the FLOT regimen has been available for our patients in the public sector, and we are looking forward to offering the survival benefit seen in the FLOT4 study to our patients.

The PRECISO trial collected quality of life data [26], blood samples and digitalised pathology slides that will serve for future studies in this field.

## Conclusions

Our study confirms with real world data, that perioperative chemotherapy is feasible in public hospitals in Chile, and should be offered as an alternative to primary surgery for patients with locally advanced GC. Observational studies like PRECISO have the potential to provide data to support the introduction of new cost-effective therapies in the standard of care in countries with health systems with limited resources.

## Trial registration

NCT0163320 at www.clinicaltrials.gov.

## Conflicts of interest

The authors have no conflicts of interest to declare.

## Funding statement

MC acknowledges partial financial support from the National Agency for Research and Development (ANID) projects ICN09_015, PIA/ACT192015; Corporación de Fomento de la Producción (CORFO; 16CTTS-66390) and Deutscher Akademischer Austauschdienst (DAAD; 57220037, 57168868).

AHC acknowledges Comisión Nacional de Investigación Científica y Tecnológica (CONICYT) - Fondo de Financiamiento de Centros de Investigación en Áreas Prioritarias (FONDAP) 1513001 and Fondo Nacional de Desarrollo Científico y Tecnológico (FONDECYT) 1191928 from the National Agency for Research and Development (ANID), Government of Chile.

## Figures and Tables

**Table 1.** Patient and tumour characteristics.

Patient and tumour characteristics	(*n* = 61)	%
Age: median (range)	62 years (23–76)	
**Sex** Male Female	44 17	72 28
**ECOG** 0 1	42 19	69 31
**Comorbidities** Hypertension Diabetes Obesity	18 7 7	30 11 11
**Laparoscopy performed** Yes No	33 28	54 46
**Site of tumour^a^** Proximal and oesophagogastric junction (cardia) Stomach (mid and distal) (non-cardia)	32 29	52 48
**Histological type** Intestinal Diffuse Indeterminate	38 9 14	62 15 23
**Presence of signet ring cells^b^** Yes No	19 42	31 69
**HER 2** Negative (0 or 1+) Equivocal (2+) Positive (3+)	*N* = 23 18 1 4	% 78 4 17

a patients with extensive tumor compromising cardia where classified as proximal

b reported on diagnostic biopsy on endoscopy

**Table 2.** Surgical treatment.

Surgical treatment	(*n* = 61)	%
Not operated	5	8
Biopsy confirmed metastatic disease	2	3
Partial gastrectomy	6	10
Total gastrectomy	46	75
Total gastrectomy and oesophagectomy	2	3
**Splenectomy** Yes No	(*n* = 54) 16 38	30 70
**Pancreatectomy** Yes No	(*n* = 54) 4 50	7 93
**Margins** Free Involved	(*n* = 54) 51 3	94 6
**Lymph node (LN) resection** Mean no of LN resected (range) <15 <25–15 25 and more	(*n* = 54) 31 (5–77) 2 10 42	4 19 78

**Table 3.** Toxicities.

Adverse events	Preoperative chemotherapy		Postoperative chemotherapy	
	(*n* = 61)	%	(*n* = 27)	%
Serious adverse events	3 1 anaemia 1 CVA^a^, 1 emesis	5	2 1 neutropenia 1 grade 3 weight loss	7
Gastrointestinal Grade 2 emesis Grade 3 emesis Grade 4 emesis Grade 2 mucositis Grade >2 mucositis Grade 2 diarrhoea Grade 3 diarrhoea Grade 4 diarrhoea	28 4 0 14 0 6 1 0	46 7 0 23 0 10 2 0	12 1 0 5 0 8 0 0	44 4 0 19 0 30 0 0
**Haematological** Grade 2 neutropenia Grade 3 neutropenia Grade 4 neutropenia Febrile neutropenia Grade 1 thrombocytopenia Grade >1 thrombocytopenia Grade 2 anaemia Grade 3 anaemia Grade 4 anaemia	15 11 1 0 1 0 4 2 0	25 18 2 0 2 0 7 3 0	5 11 2 0 0 0 3 0 0	19 41 7 0 0 0 11 0 0
**Skin** Grade 1 hand foot syndrome Grade 2 hand foot syndrome Grade 3 hand foot syndrome Grade 1 alopecia Grade 2 alopecia	15 9 0 27 12	25 15 0 44 20	5 2 0 9 0	19 7 0 33 0
**Constitutional** Grade 1 fatigue Grade 2 fatigue Grade 3 fatigue Grade 1 weight loss Grade 2 weight loss Grade 3 weight loss	14 15 2 12 1 0	23 25 3 20 2 0	9 7 0 6 4 1	33 26 0 22 15 4

a Cerebrovascular accident

**Table 4.** Clinical (pre-treatment) and pathological (post-treatment) staging.

Staging	Pretreatment (cTNM^b^)		At surgery (pTNM^c^)	
	(*n* = 61)	%	(*n* = 56)	%
Tx	21	34	2	4
T<3	11	18	33	59
T3	26	43	19	34
T4	3	5	2	4
N0	8	13	18	32
N+	38	62	36	64
Nx	25	41	2	4
M0	61	100	54	96
M1	0	0	2	4
pCR^a^ (pT0N0)			2	4

a pCR = pathological complete response

b cTNM = clinical tumour node metastasis stage

c pTNM = pathological tumour node metastasis stage

**Table 5.** Multivariable analysis.

Parameter	Univariate (p-value)	HR (95% CI)
Laparoscopy Signet cell cT cN pT pN	0.179 0.157 0.924 0.699 0.143 0.008	0.65 (0.34 – 1.0) 1.63 (0.83 – 3.0) 1.03 (0.55 – 1.95) 0.88 (0.46 – 1.69) 1.68 (0.84 – 3.34) 3.42 (1.41 – 8.34)

## References

[1] Ferlay J, Ervik M, Lam F (2018). Global Cancer Observatory: Cancer Today.

[2] GBD 2017 Stomach Cancer Collaborators (2020). The global, regional, and national burden of stomach cancer in 195 countries, 1990-2017: a systematic analysis for the global burden of disease study 2017. Lancet Gastroenterol Hepatol.

[3] Heise K, Bertran E, Andia ME (2009). Incidence and survival of stomach cancer in a high-risk population of Chile. World J Gastroenterol.

[4] García CC, Benavides CC, Apablaza SP (2007). Resultados del tratamiento quirúrgico del cáncer gástrico: Análisis de 423 casos [Surgical treatment of gastric cancer: results in 423 cases]. Rev Med Chil.

[5] Macdonald JS, Smalley SR, Benedetti J (2001). Chemoradiotherapy after surgery compared with surgery alone for adenocarcinoma of the stomach or gastroesophageal junction. N Engl J Med.

[6] Sakuramoto S, Sasako M, Yamaguchi T (2007). Adjuvant chemotherapy for gastric cancer with S-1, an oral fluoropyrimidine. N Engl J Med.

[7] Cunningham D, Allum WH, Stenning SP (2006). Perioperative chemotherapy versus surgery alone for resectable gastroesophageal cancer. N Engl J Med.

[8] Ychou M, Boige V, Pignon JP (2011). Perioperative chemotherapy compared with surgery alone for resectable gastroesophageal adenocarcinoma: an FNCLCC and FFCD multicenter phase III trial. J Clin Oncol.

[9] Müller B, Balbontín P, Cárcamo M (2009). Quimiorradioterapia adyuvante en el cáncer gástrico resecado con intención curativa: Análisis de supervivencia y toxicidad de pacientes tratados entre 1995 y 2003 en el Instituto Nacional del Cáncer, Chile [Results of adjuvant chemoradiation after curative surgery for gastric cancer: a retrospective study]. Rev Med Chil.

[10] Bossert TJ, Leisewitz T (2016). Innovation and change in the chilean health system. N Engl J Med.

[11] OECD Reviews of Public Health (2019). Chile: A Healthier Tomorrow.

[12] Greene F L, American Joint Committee on Cancer (2002). Digestive System: Stomach. AJCC Cancer Staging Manual.

[13] Oken MM, Creech RH, Tormey (1982). Toxicity and response criteria of the Eastern Cooperative Oncology Group. Am J Clin Oncol.

[14] (2003). Cancer Therapy Evaluation Program, common terminology criteria for adverse events, version 3.0, DCTD, NCI, NIH, DHHS. https://ctep.cancer.gov/protocoldevelopment/electronic_applications/docs/ctcaev3.pdf.

[15] MATLAB (2014). MathWorks Introduces Major New Release of MATLAB.

[16] Homann N, Pauligk C, Luley K (2012). Pathological complete remission in patients with oesophagogastric cancer receiving preoperative 5-fluorouracil, oxaliplatin and docetaxel. Int J Cancer.

[17] Smyth EC, Fassan M, Cunningham D (2016). Effect of pathologic tumor response and nodal status on survival in the medical research council adjuvant gastric infusional chemotherapy trial. J Clin Oncol.

[18] Al-Batran SE, Homann N, Pauligk C (2019). Perioperative chemotherapy with fluorouracil plus leucovorin, oxaliplatin, and docetaxel versus fluorouracil or capecitabine plus cisplatin and epirubicin for locally advanced, resectable gastric or gastro-oesophageal junction adenocarcinoma (FLOT4): a randomised, phase 2/3 trial. Lancet.

[19] Biffi R, Fazio N, Luca F (2010). Surgical outcome after docetaxel-based neoadjuvant chemotherapy in locally-advanced gastric cancer. World J Gastroenterol.

[20] Xiong BH, Cheng Y, Ma L (2014). An updated meta-analysis of randomized controlled trial assessing the effect of neoadjuvant chemotherapy in advanced gastric cancer. Cancer Invest.

[21] Norero E, Vega EA, Diaz C (2017). Improvement in postoperative mortality in elective gastrectomy for gastric cancer: analysis of predictive factors in 1066 patients from a single centre. Eur J Surg Oncol.

[22] Cats A, Jansen E, van Grieken N (2018). Chemotherapy versus chemoradiotherapy after surgery and preoperative chemotherapy for resectable gastric cancer (CRITICS): an international, open-label, randomised phase 3 trial. Lancet Oncol.

[23] Kuhnle PJ, Israel KF, Menges M (2019). Real-life data on improvement of survival after perioperative chemotherapy versus surgery alone on resectable adenocarcinoma of the stomach – a single-center study. Real-life-daten zur Verbesserung des Überlebens nach perioperativer chemotherapie im Vergleich zu alleiniger operation beim resektablen Magenkarzinom – eine single-center-studie. Z Gastroenterol.

[24] Yoshikawa T, Tanabe K, Nishikawa K (2014). Accuracy of CT staging of locally advanced gastric cancer after neoadjuvant chemotherapy: cohort evaluation within a randomized phase II study. Ann Surg Oncol.

[25] Martínez Lago N, Vieito Villar M, Varelo Ponte R (2020). Impact of HER2 status in resected gastric or gastroesophageal junction adenocarcinoma in a Western population. Ecancer.

[26] Betancour P, Müller B, Sola J (2019). Quality of life and preoperative chemotherapy in gastric cancer in Chile: results from the observational study of perioperative chemotherapy in gastric cancer (PRECISO). Ann Oncol.

